# How Fear of External Threats Plays Roles: An Examination of Supervisors’ Trait Anger, Abusive Supervision, Subordinate Burnout and CCB

**DOI:** 10.3390/ijerph192416810

**Published:** 2022-12-14

**Authors:** Wen Zhang, Wei Liu, Yingyee Wu, Chenlu Ma, Xiyao Xiao, Xichao Zhang

**Affiliations:** 1 Faculty of Psychology, Beijing Normal University, Beijing 100875, China; 2 Faculty of Industrial Design Engineering, Technical University Delft, 2628 CE Delft, The Netherlands

**Keywords:** abusive supervision, burnout, compulsory citizenship behaviors, fear of external threats, trait anger

## Abstract

In times of uncertainty, such as during COVID-19, many organizations experience profit decline, and employees develop a fear of external threats, such as organizational layoffs. However, most of the literature focuses on how people’s fear influences their well-being. Less is known about how employees’ fear of external threats influences their workplace behaviors. The current study proposes that supervisors’ fear of external threats stimulates those who are high in trait anger to behave in a more abusive way. Simultaneously, subordinates’ fear of external threats would strengthen the positive relationship between abusive supervision and their burnout and compulsory citizenship behaviors (CCB), as fear of external threats constrains their response options to abusive supervision. We tested the hypotheses with a multiwave and multisource survey study (*N* = 322 dyads) in China, and the results showed that supervisors’ fear of external threats strengthened the positive effect of trait anger on abusive supervision. Subordinates’ fear of external threats strengthens the positive relationships of abusive supervision with CCB and the mediating effect of abusive supervision in the relationship of supervisors’ trait anger with subordinates’ CCB. Our study enriches people’s understanding of how supervisors’ and subordinates’ fear of external threats may play roles in workplace behaviors.

## 1. Introduction

Employees’ fear of external threats refers to negative psychological emotions that involve uncertainty and danger from undesirable events or harm from outside of the organization [1]. Typical sources of external threats include economic or industry turndown, competitive threats, changes in technology, or mergers and acquisitions ([1]). Since the outbreak of the COVID-19 pandemic in China and globally, profits have declined sharply in many industries and organizations, and a large number of firms have been bankrupted [2]. Many employees suffer from the uncertainty of external threats. Employees may worry about the survival of their organizations and the security of their jobs. Although layoffs happen within the organization, they pose greater risks and uncertainty to employees’ careers, income, and financial status. Previous literature mainly focuses on how people’s fear of uncertainty or threat potentially influences their well-being and lives [3,4]; less is known about how employees’ fear of external threats influences both supervisors’ and employees’ behaviors in the workplace [5,6]. The current study intends to fill this gap by exploring how supervisors’ fear of external threats relates to their enaction of abusive supervision and how subordinates’ fear of external threats is related to their responses to abusive supervision.

Abusive supervision is defined as “subordinates’ perception of the extent to which supervisors engage in the sustained display of hostile verbal and nonverbal behaviors, excluding physical contact” [7]. Drawing on trait activation theory [8,9], which posits that personality should be examined in terms of situation-behavior profiles [10], the current study proposes that supervisors’ trait anger is activated by fear of external threats, which then leads to more abusive supervision. Previous studies have shown that individuals with higher levels of trait anger are more likely to be irritated and experience state anger [11]. Fear of external threats, such as an uncertain future of the organization, negative impacts of the economic turndown, and organizational layoffs, disturbs them, which pushes them to behave in a more aggressive and abusive way toward their subordinates.

**Figure 1:**
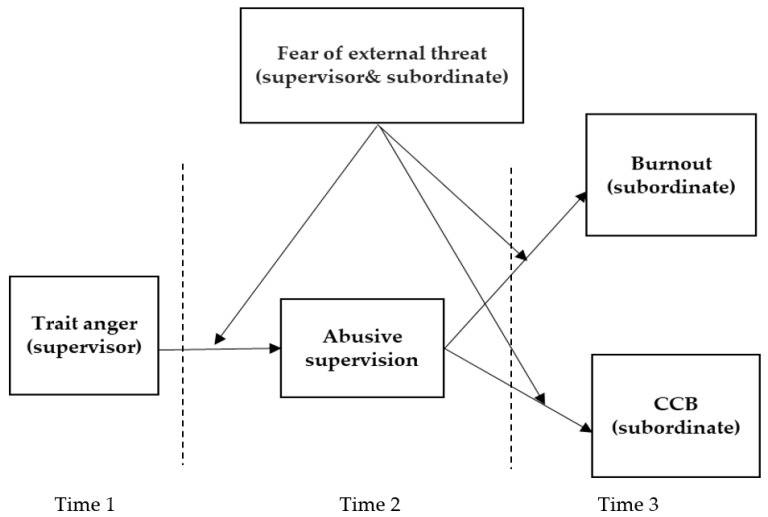
Theoretical Model. Dash line represents data collection at different time points.

Switching the perspective from supervisors to subordinates, we propose that subordinates’ fear of external threats exacerbates the relationships of abusive supervision with their well-being (i.e., burnout) and maladaptive work behaviors (compulsory citizenship behaviors). As proposed by Oh and Farh [12], the environment constrains subordinates’ responses to abusive supervision. On the one hand, as abusive supervision itself is a severe stressor [7], fear of external threats adds to subordinates’ stress and leads to higher levels of burnout [13]. On the other hand, fear of external threats implies that abused subordinates are less likely to choose costly or risky behaviors such as turnover or retaliation [12]; they may succumb to supervisors’ abusive power by engaging in extra-role behaviors against their will, which is termed compulsory citizenship behaviors (CCB, [14]). Figure 1 demonstrates the conceptual model of the current study.

The current study contributes to the literature in three ways. First, we examine how the fear of external threats in times of uncertainty influences both supervisors’ and subordinates’ workplace behaviors. Such exploration is largely understudied but important, as it illustrates employees’ psychological and behavioral responses in the workplace. Second, the current study adds to the abusive supervision-related literature by extending the antecedent and the condition of the antecedent. Previous literature usually adopts social learning [15,16], self-regulation impairment [17,18], moral exclusion [19,20], and justice perspectives [21] to explain the occurrence of abusive supervision. Drawing on trait activation theory [8,9], the current study adds another perspective by revealing that supervisors’ fear of external threats may potentially activate their trait anger and give rise to abusive supervision. Last, although most previous studies have examined the positive relationship between abusive supervision and burnout, in addition to well-being, we also investigate supervisors’ CCB as a relatively novel and important outcome [22] and further illustrate under which conditions abused subordinates are more likely to engage in CCB in response to abusive supervision.

## 2. Theoretical Background and Hypothesis Development

### 2.1. Moderating Effect of Supervisors’ Fear of External Threats

According to trait activation theory [8,9], personality should be examined in situation-behavior profiles, which capture personality-driven behaviors across different situations [10]. In other words, trait activation theory suggests that if a person has the personality of X and when he or she is exposed to a particular situational cue, he or she is likely to respond in particular and predictable ways [10]. In accordance with trait activation theory [8], we identify supervisors’ fear of external threats as a special situational cue that triggers an inherently angry supervisor’s inclination toward abusive supervision.

Trait anger is the tendency to perceive a wide range of environments as anger-provoking [23]. It is a stable personality dimension of anger proneness. Supervisors with higher levels of trait anger are more easily provoked and more prone to experience anger [23]. Previous literature has shown that trait anger is positively related to interpersonal conflict, attitudes toward revenge and hostility, and procedural injustice in the workplace [24]. In the context of supervisors, when they have higher levels of trait anger, they may capture interpersonal aggression more easily from upper managers, subordinates, or customers [25,26] and then vent their anger toward relatively safe targets, in this case, subordinates who have less hierarchical power [27,28]. Indeed, prior literature has established a positive relationship between supervisors’ trait anger and abusive supervision (e.g., [16,29]).

We further propose that supervisors’ fear of external threats strengthens the positive relationship between trait anger and abusive supervision. In normal times, individuals with high trait anger may use their anger and its accompanying energy to achieve competitive success or to gain a dominant position in the working system (e.g., [10]). However, in times of uncertainty, many companies are faced with severe challenges and even survival issues. For example, hotels, travel, and restaurants are barely able to continue their businesses [2]. For supervisors with higher trait anger, when they are under the fear of uncertainty, layoffs, reduced profits or a recessionary industry, their emotions are in a highly aroused state, and, thus, they are more likely to be irritated and enraged [11]. Their trait of anger may be activated by their fear of external threats and then transform into abusive behaviors such as picking fault with subordinates and undermining, ridiculing and telling subordinates that they are incompetent.

**Hypothesis** **1:** *Supervisors’ fear of external threats moderates the positive relationship between their trait anger and abusive supervision* *; this relationship is more positive when supervisors’ fear of external threats is at a higher level (vs. at a lower level).*

### 2.2. Subordinates’ Burnout and Compulsory Citizenship Behavior in Response

Burnout, as an important indicator of employees’ well-being [13], is defined along three dimensions: emotional exhaustion, depersonalization or cynicism, and diminished personal accomplishment [30]. As noted by Rifkin et al. [31], individuals pay particular attention to negative events that interfere with their lives. They may ruminate over the event and have negative emotions, all of which would consume and drain their resources [31] and thus lead to higher levels of burnout. Specifically, when employees experience abusive supervision, such as public undermining and ridiculing, they feel negative emotions and regulate their resources to cope with the abusive behavior as well as their own negative emotions. These processes lead to employees’ higher levels of burnout. Previous literature has indicated a positive relationship between abusive supervision and employee burnout (for a review, see [32]).

In addition to well-being, abusive supervision may further relate to employees’ workplace behavior, such as decreased organizational citizenship behavior (i.e., OCB, [33]), defined as “individual behavior that is discretionary, not directly or explicitly recognized by the formal reward system, and that in aggregate promotes the effective functioning of the organization” [34]. OCB includes extra-role behaviors such as interpersonal helping behaviors that are not included in job descriptions [35]. According to Vigoda [35], there is a fine line between individuals’ genuine, spontaneous “goodwill” (i.e., OCB) and their response to external pressure by significant others in the workplace, such as supervisors or coworkers (i.e., CCB); in other words, they feel they must do something because refusing to do so is not an option.

CCB can be viewed as a means by which those in power take advantage of others because those who are less powerful sometimes cannot resist or say no to those who have more power and who are in higher positions [22]. When employees experience abusive supervision, such as enforcing, undermining, and yelling, subordinates respond with retaliation, such as decreased OCB, withdrawal, and deviant behaviors ([7]). However, as noted by Zellars et al. [36], although abusive supervision and OCB are moderately and negatively related, abusive supervision may be positively related to CCB. This notion is further theoretically and empirically supported by Vigoda-Gadot [14] and Zhao et al. [22]. According to Tepper et al. [37], supervisors use abusive supervision to discipline subordinates and adjust their subordinates’ deviant behaviors. Similarly, supervisors may also adopt abusive supervision to encourage subordinates to behave compliantly and engage in higher levels of OCB. Vigoda-Gadot [14] suggested that as higher levels of OCB usually indicate a healthier and more successful organization, supervisors encourage their subordinates to enact OCB; however, the pressure to reach higher levels of OCB may also increase the possibility that supervisors use other means, such as abusiveness and exploitation. Additionally, to avoid abusive supervision in the future, subordinates may show their compliance and display forced OCB, in this case, CCB.

Integrating those proposals, we argue that supervisors’ trait anger may have far-reaching effects on subordinates’ burnout and CCB. When supervisors have higher levels of trait anger, they are more likely to be irritated and vent their anger or aggression to relatively safe targets—their subordinates (e.g., [10,28]). Subordinates who experience abusive supervision may indulge in negative emotions and events and experience a resource loss spiral and, thus, feel higher levels of burnout (e.g., [13]). Simultaneously, due to power and position differences, abused subordinates are unlikely to retaliate directly against their supervisors because such behaviors are less likely to hurt the supervisors but jeopardize their own positions in the organizations (e.g., [36,38]).

**Hypothesis** **2:** *Abusive supervision mediates the positive relationships of supervisors’ trait anger with subordinates’ burnout (2a) and CCB (2b)*.

### 2.3. Moderating Roles of Subordinate’s Fear of External Threat

Furthermore, as suggested by Oh and Farh [12], subordinates’ responses to abusive supervision are greatly influenced by situational factors such as power dependencies, environmental climate, and subordinates’ appraisal of their own coping ability [12,39]. For example, when employees have more work opportunities and thus less dependency on the supervisor or the organization, they can choose to leave the abusive supervisor [28,40]). Similarly, subordinates who are central to the accomplishment of work [41] or who have rare expertise that others do not have worry less about unemployment or layoffs and thus can cope better with abusive supervision [12]. In contrast, when subordinates are worried about losing jobs, they are more likely to act cautiously when faced with abusive supervision.

The current study proposes that subordinates’ fear of external threats would exacerbate the influence of abusive supervision on subordinates’ burnout. First, abusive supervision may trap subordinates in a negative state and damage their well-being, as illustrated by increased burnout [28]. Simultaneously, in times of uncertainty and economic turndown, some employees may also worry about external threats, such as the negative impact of industry turndown on organizations, the loss of revenues or sales, and the possibility of losing their jobs [42]. In the time of COVID-19, those worries become realistic as some companies have closed, and some industries continue layoffs [43]. Previous literature has shown the negative influences of layoff experiences and job insecurity on employees’ well-being (e.g., [44,45]). On the one hand, subordinates’ fear of external threats may add pressure in addition to abusive supervision, giving rise to higher levels of burnout. On the other hand, fear of external threats may force subordinates to tolerate abusive supervision without further retaliation, draining subordinates’ personal resources and leading to higher levels of burnout.

In a similar vein, we posit that subordinates’ fear of external threats also strengthens the positive relationship between abusive supervision and employees’ CCB. In the theoretical framework proposed by Oh and Farh [12], subordinates’ coping strategies may be constrained by environmental factors. In the current situation, subordinates also take the influence of COVID-19, the unemployment rate, and layoffs into consideration. For subordinates with higher levels of fear of external threats, if they leave the organization or retaliate against their supervisors, which may negatively affect their positions in the organization and run the risk of being laid off [12], they cannot expect to easily find another job (e.g., [4,46]). Moreover, leaving the organization without an alternative job option means financial burden and personal pressure, which makes it a costly action. Therefore, when faced with abusive supervision, those who have higher levels of fear of external threats are more likely to invest extra effort to show their value or commitment to the organizations, even unwillingly.

In this respect, we further propose that subordinates’ fear of external threats moderates the mediating effects of abusive supervision in the relationships of supervisors’ trait anger with subordinates’ burnout and CCB. For subordinates who are more worried about the uncertainty of the external environment and possible layoffs, they may tolerate the supervisors’ aggressive behaviors resulting from trait anger. When supervisors are irritated by other stressful or threatening factors, they may vent their anger to their subordinates [27,28]. Subordinates who suffer from the stress of abusive supervision, as well as the influences of external threats, will develop higher levels of burnout [13]. Abused subordinates may also regulate their behaviors by conducting more CCB that is superficially beneficial to the organizations or the supervisors to avoid further negative consequences [22].

**Hypothesis** **3:** *Subordinates’ fear of external threats moderates the positive relationship between abusive supervision and subordinates’ burnout (3a) and CCB (3b); such relationships are more positive when subordinates’ fear of external threats is at a higher level (vs. lower level)*.

**Hypothesis** **4:** *Subordinates’ fear of external threats moderates the mediating effects of abusive supervision in the relationship between supervisors’ trait anger and subordinates’ burnout (4a) and subordinates’ CCB (4b); such mediating effects are stronger when subordinates’ fear of external threats is at a higher level (vs. lower level)*.

## 3. Method

### 3.1. Sample and Procedure

We collected data at 3 time points with a one-month interval between February and April 2022 in China. Tens of thousands of tech employees were laid off from July 2021 to April 2022 [24]. Additionally, industries such as tourism and restaurants have not fully recovered from COVID-19. Therefore, we believe it is a critical time point to conduct the study. The current study adopts a 3-wave prospective study design because appropriate time lags may help reduce common method problems [21]. In accordance with previous studies (e.g., [33,47]), we chose one month as the interval. If the time lag is too short, it cannot reduce the salience of the predictor variable. However, if the time lag is too long for the theoretical relations, respondents’ attribution may be problematic, and it may thus mask a relationship that really exists [21].

As the current study involves both supervisors and subordinates, we recruited dyads (i.e., supervisor and subordinate) to participate in our study. We mailed surveys to supervisors who were randomly selected from a multi-occupation database through an online survey company. Supervisors and subordinates had previously registered in the participant pool, and we chose dyads whose response acceptance rates were higher than 80% to ensure the survey quality. Additionally, we set rules that one participant with 1 IP address could fill in the survey once, and each participant had to provide the last 4 numbers of their phone number for future matching, and if there were repetitive phone numbers that were provided by participants, we only selected 1 and excluded the others from further analyses. Furthermore, as every participant would be matched with his or her supervisor or subordinate, it is less likely that 1 subject answered a questionnaire multiple times.

We randomly sent survey links to the supervisors in the participation pool and asked them to provide at least one of their subordinates’ contact information. At time 2, we randomly sent information to the subordinates and invited them to fill in the surveys. At time 3, we invited the same subordinates who took part in the second wave’s survey. All participation was anonymous and confidential. Participants could only continue filling in questionnaires before giving their informed consent. Approval for the study was obtained from the ethics committee of the first and second authors’ university. All procedures used in this study adhered to the tenets of the Declaration of Helsinki. Questionnaires included a cover letter that indicated the purpose of the study and assured confidentiality of the participants’ answers. Participants who agreed with the statement could choose to continue completing the following questionnaires.

At time 1 (T1), we received 610 supervisors’ responses to the survey that measured supervisors’ trait anger and fear of external threats. Approximately one month later, at T2, we randomly sent survey links to one of their subordinates to measure subordinates’ perceptions of abusive supervision and their fear of external threats. We received 443 returned responses. Another month later, at T3, we sent the same subordinates the survey about their burnout and CCB. We received 389 returned responses. Each participant was rewarded with 5 RMB ($0.695) for completing the questionnaire at each time point. For subordinates who completed 2 surveys, they had a chance to win a lottery with a prize of 166 RMB ($23.057).

### 3.2. Measures

Standard translation and back-translation procedures were followed to ensure that all the items in the questionnaires were accurately translated from English to Chinese [48].

Trait anger was measured with the ten-item Trait Anger Scale (TAS) developed by Spielberger [49]. This scale consists of 2 dimensions: angry temperament, referring to the disposition to experience anger without provocation (i.e., “I am a hotheaded person”), and angry reaction, measuring the frustrations or negative evaluations that may be related to situational provocations (i.e., “I get angry when I’m slowed down by others’ mistakes”). Participants were asked to rate from 1 (“almost never”) to 4 (“almost always”) to indicate how angry they generally felt. Cronbach’s alpha was 0.88 for the angry temperament dimension and 0.84 for the angry reaction dimension. Cronbach’s alpha of the overall scale of trait anger was 0.90.

Fear of external threats was measured with a five-item Fear of External Threat at Work Scale developed by [1]. An example item was, “during the last several months, I have felt fear that there would be layoffs at this organization.” Both supervisors and subordinates were asked to rate from 1 (“not at all”) to 5 (“very often”) to indicate their fears. Cronbach’s alpha was 0.85 for supervisors’ fear of external threats and 0.82 for subordinates’ fear of external threats in the current study.

Abusive supervision was measured with a Chinese version of the 15-item Abusive Supervision Scale of Tepper [9], which was translated and verified by Yang et al. [50]. An example item was “My supervisor puts me down in front of others.” Subordinates were asked to rate from 1 (“never”) to 5 (“always”) to indicate the frequency of abusive supervision. Cronbach’s alpha was 0.89 in the current study.

Burnout was measured by the 16-item MBI-GS [30]. It consists of three dimensions: emotional exhaustion (e.g., “I feel emotionally drained from my work”), cynicism (e.g., “I have become less enthusiastic about my work”), and professional efficacy (e.g., “I feel I am making an effective contribution to what this organization does”). All items were scored on a 6-point scale, ranging from 1 (“strongly disagree”) to 6 (“strongly agree”). In the analysis, we reversed the professional efficacy score. Cronbach’s alpha was 0.87 for the emotional exhaustion dimension, 0.83 for the cynicism dimension, and 0.84 for the professional efficacy dimension. Cronbach’s alpha of the overall scale of burnout was 0.89.

Compulsory citizenship behavior (CCB) was measured with the five-item CCB scale of Vigodagadot [14]. An example item was, “I feel that I am expected to invest more effort in this job than I want to and beyond my formal job requirements”. Participants were asked to rate from 1 (“never”) to 5 (“always”) to show how frequently they conduct CCB. Cronbach’s alpha was 0.88 in the current study.

## 4. Results

### 4.1. Analytical Strategy

First, we conducted confirmatory factor analyses (CFAs) with Mplus 7.4 [51] to examine whether the measurement scales represented distinct constructs. As trait anger has two dimensions and burnout consists of three dimensions, we conducted second-order CFAs with two first-order factors loaded on one trait anger factor and three first-order factors loaded on one burnout factor. Then, we compared the measurement fit of our hypothesized six-factor model with alternative models that have fewer factors. Furthermore, we employed path analysis with maximum likelihood estimation in Mplus to test the hypotheses. First, in Model 1, we used the XWITH command to construct integration to test the moderation effect of supervisors’ trait anger with fear of external threats on abusive supervision. Specifically, we centered variables included in the interaction terms to reduce multicollinearity. In Model 2, we used bootstrapping with 10,000 iterations to test the mediating effect of abusive supervision on the relationship of trait anger with subordinates’ burnout and CCB. Next, in Model 3, on the basis of Model 2, we entered the interaction term to simultaneously test the moderation effect of subordinates’ fear of external threats and abusive supervision on CCB and burnout and the moderated mediation effect of abusive supervision in the relationship between supervisors’ trait anger with subordinates’ CCB and burnout. Finally, we addressed the common method issue and tested an alternative model to ensure the robustness of our model.

### 4.2. Confirmatory Factor Analysis

As shown in Table 1, the hypothesized six-factor (i.e., supervisors’ trait anger, supervisors’ fear of external threats, abusive supervision, subordinates’ fear of external threats, burnout, and CCB) model exhibited a good fit to the data (χ^2^(604) = 996.17, *p* < 0.001, SRMR = 0.04, CFI = 0.95, TLI = 0.94, and RMSEA = 0.05). This model was superior to other alternative models, such as combining two outcomes together and combining all items into one factor.

### 4.3. Descriptive Statistics

After matching three-wave and supervisor and subordinates’ responses with a unique code and removing participants who had at least one of the attention checks items wrong (“please choose 4 for this item”), the final sample consisted of 322 dyads. Of the supervisor sample, 59.3% were male, and their average age was 36.21 years old (*SD* = 7.51). Of the subordinate sample, approximately 42.5% were male, and their average age was 27.93 years (*SD* = 7.01). The supervisors’ and subordinates’ average dyadic working tenure was 5.98 years (*SD* = 3.78). Most of the participants (56.5%) were from the high-tech industry, approximately 26.4% were from the service industry, approximately 9.2% were from the financial industry, and 3.6% were from the education industry. 

Table 2 shows the means, standard deviations, and correlations of the studied variables.

### 4.4. Hypothesis Testing

**Figure 2:**
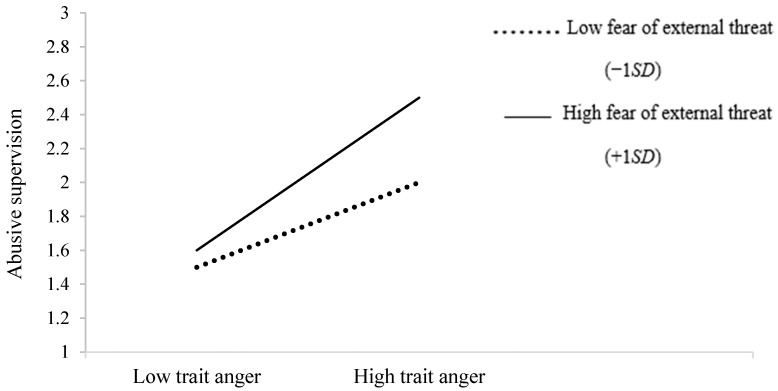
The moderating role of supervisors’ fear of external threats in the relationship between trait anger and abusive supervision.

We tested our hypotheses with path analyses in Mplus. In support of Hypothesis 1, the result of Model 1 showed that supervisors’ fear of external threats interacted with trait anger to predict abusive supervision (*β* = 0.20, *p* < 0.001). Specifically, as shown in Figure 2, the relationship was more positive when supervisors experienced higher levels (+1 *SD*) of fear of external threats (*β* = 0.45, *p* < 0.001) rather than lower levels (−1 *SD*; *β* = 0.18, *p* = 0.001). Additionally, as shown in Table 3 (Model 2), the bootstrapping result showed that abusive supervision mediated the positive relationship of supervisors’ trait anger with subordinates’ burnout (indirect effect = 0.08, 95% CI = [0.039, 0.126]), and with CCB (indirect effect = 0.10, 95% CI = [0.048, 0.171]). The mediating effect explained 37.2% of the variance in burnout and 14.1% of the variance in CCB. Therefore, Hypotheses 2a and 2b were supported.

**Figure 3:**
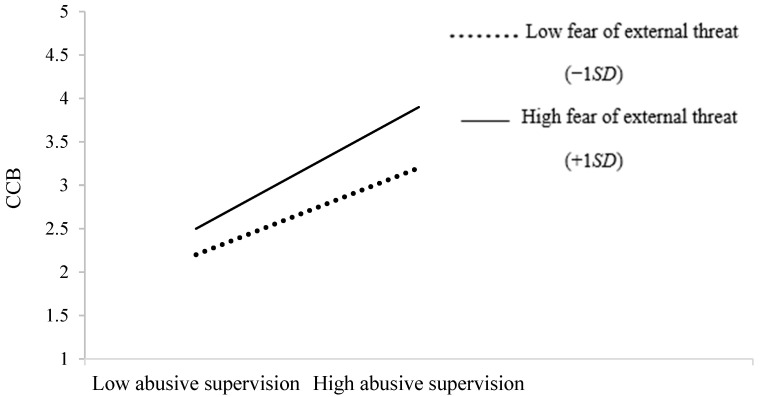
The moderating role of subordinates’ fear of external threats in the relationship between abusive supervision and CCB. Note: CCB = compulsory citizenship behaviors.

Hypothesis 3 proposed that subordinates’ fear of external threats would moderate the positive relationship of abusive supervision with subordinates’ burnout (3a) and with subordinates’ CCB (3b). As illustrated in Model 3 of Table 3, subordinates’ fear of external threats did not moderate the relationship between abusive supervision and subordinates’ burnout (*β* = 0.03, *p* = 0.696), but it strengthened the positive relationship between abusive supervision and subordinates’ CCB (*β* = 0.23, *p* < 0.001). Specifically, the relationship of abusive supervision with CCB was more positive (*β* = 0.33, *p* = 0.006) when subordinates had a 1 *SD* higher level of fear of external threats than *a* 1 *SD* lower level of fear of external threats (*β* = 0.80, *p* < 0.001; see Figure 3); thus, Hypothesis 3a was not supported, but Hypothesis 3b was supported.

Furthermore, we did not find a moderated mediating effect of abusive supervision on the relationship between supervisors’ trait anger and subordinates’ burnout (*β* = 0.01, 95% CI = [−0.004, 0.005]). However, subordinates’ fear of external threats moderated the mediating effect of abusive supervision in the relationship between supervisors’ trait anger and the CCB (*β* = 0.10, 95% CI = [0.038, 0.165]). Specifically, the mediating effect of abusive supervision was more positive at higher levels (+1 *SD*) of subordinates’ fear of external threats (indirect effect = 0.17, 95% CI = [0.095, 0.247]) than at lower levels (−1 *SD*; indirect effect = 0.03, 95% CI = [−0.037, 0.100]). The moderated mediating effect accounted for 45.3% of the variance in subordinates’ burnout and 30.1% of CCB. Therefore, Hypothesis 4a was not supported, and Hypothesis 4b was fully supported.

### 4.5. Supplemental Analyses

To ensure the robustness of our model, we conducted several supplemental analyses. First, we addressed the common method issue by creating a new construct named the common latent factor (e.g., [52]). The results showed that the differences in the standardized regression weights of the constraint and unconstrained models were smaller than 0.20 for all the latent variables. Therefore, we assume that the current results were not substantially contaminated by common method bias.

Additionally, we reran the model without control variables, and the results remained unchanged regarding the directions and significance of the coefficients. Specifically, we found that supervisors’ fear of external threats positively moderated the relationship of trait anger with abusive supervision (*β* = 0.19, *p* < 0.001); abusive supervision mediated the relationship of supervisors’ trait anger with subordinates’ burnout (indirect effect = 0.07, 95% CI = [0.032, 0.121]) and CCB (indirect effect = 0.11, 95% CI = [0.056, 0.182]). Subordinates’ fear of external threats moderated the positive relationship between abusive supervision and subordinates’ CCB (*β* = 0.31, *p* = 0.002) but not burnout (*β* = 0.03, *p* = 0.661). Furthermore, subordinates’ fear of external threats also moderated the mediating effect of abusive supervision in the relationships of trait anger with subordinates’ CCB (*β* = 0.09, 95% CI = [0.032, 0.156]), but not with burnout (*β* = 0.01, 95% CI = [−0.035, 0.054]).

## 5. Discussion

Based on trait activation theory [8,9] and the literature on the response to abusive supervision based on cognitive appraisals [12,39], our study proposed how supervisors’ and subordinates’ fear of external threats would influence their enactment of and response to abusive supervision, respectively. Specifically, with a multiwave and multisource survey study, the current study found that supervisors’ fear of external threats acts as an environmental stimulus to activate supervisors’ trait anger and thus relates to more abusive supervision. Furthermore, subordinates’ fear of external threats strengthens their CCB reactions to abusive supervision. In other words, subordinates who are more worried about their organizational survival or layoff issues respond to abusive supervision by engaging in extra-role behaviors, despite those behaviors being against their will.

However, we did not find a moderating effect of subordinates’ fear of external threats in the relationship of abusive supervision with subordinates’ burnout. We speculated that this might be because subordinates may experience worry, anxiety or depression, but such negative emotions may not necessarily equate with negative (e.g., cynical) attitudes toward their work. In contrast, external threats may potentially make subordinates value their current work more, and such an effect may counterbalance the negative effect of abusive supervision on subordinates’ burnout.

### 5.1. Theoretical Implications

Our study has some theoretical implications. First, it illustrates, in uncertain circumstances, how employees’ fear of external threats plays a role in their organizational behaviors. Most previous literature mainly focused on how people’s fear of uncertainty would influence their well-being or psychological health [3]. Fewer studies have paid attention to how employees’ fear of external threats may influence their workplace behaviors [2]. Considering that work is one of the most important dimensions of people’s lives, it deserves a deep investigation as there are a variety of emotions often experienced at work, including fear. Recent studies have shown that employees’ fear of external threats at uncertain times leads to increased silence and decreased creativity, and decreased organizational citizenship behavior [2,53]. The current study extends people’s understanding of the influence of employees’ fear of external threats. Specifically, the current study integrated perspectives from supervisors and subordinates and found different roles of their fear of external threats. The fear of external threats might magnify the power distances by amplifying supervisors’ inherently angry traits and simultaneously signifying the disadvantageous position of subordinates.

Additionally, drawing on trait activation theory [8], the current study adds knowledge about the antecedents and possible explanations of the occurrence of abusive supervision. As noted by Tepper [28], most previous studies have examined the antecedents of abusive supervision from the perspectives of resource depletion [17], justice [21], social learning [15,16], and moral exclusion [19,20]. The current study mainly investigated how supervisors’ traits, which should be obvious but less examined, are activated by situational cues and then relate to higher levels of abusive supervision. Previous studies have pointed out several situational factors, such as instrumental climate and power, would make Machiavellian leaders enact abusive supervision [4], and the current study identifies another important personality trait (i.e., trait anger) as well as an environmental factor (i.e., fear of external threat) that relate to abusive supervision. These findings enrich people’s knowledge regarding under which conditions abusive supervision is more likely to occur.

Furthermore, by proposing and examining the positive relationship of abusive supervision with subordinates’ CCB, the current study enriched people’s understanding of how subordinates react to abusive supervision. Previous literature has shown that abusive supervision is negatively related to organizational citizenship behaviors (OCB; [33]), but fewer studies have noted that abusive supervision also makes subordinates engage in organizational citizenship behaviors against their will [22]. Our study empirically supported this relationship and found that subordinates’ fear of external threats strengthened the positive relationship between abusive supervision and CCB. The current findings implied that subordinates’ reactions might be more obscure and less obvious, as found in a previous study (i.e., turnover, withdrawal, or deviant behaviors; [28]). Subordinates’ reactions may be constrained by the situations and their appraisals of the current situation. When they are fearful of external threats, implying that the environment or their current situation is less optimistic, they are more likely to respond to abusive supervision by enacting unwilling citizenship behaviors.

### 5.2. Practical Implications

The current study found that trait anger reinforces abusive supervision. To avoid the occurrence of abusive supervision, organizations should solve the problem from the root. When promoting employees to supervisor positions, screening of personality and leadership training should be provided to ensure that employees are appropriate for their positions [54]. For supervisors who are at a high level of trait anger, extra intervention programs such as anger management and conflict management programs should be arranged. Cognitive-behavioral techniques such as psychoeducation, cognitive reconstructing and relaxation techniques have been found to be effective in controlling anger [55]; thus, organizations should adopt techniques to train their supervisors to control anger.

Second, our study found that abusive supervision is positively related to employee burnout and CCB. To avoid the loss of the organization and subordinates, organizations should implement protective policies through rigid rules and regulations [56]. For instance, punishments should be expressly stipulated for supervisors who practice inappropriate supervision behavior. On the other hand, awareness of resisting abusive supervision should be raised among employees. Employees should learn to identify when they or their colleagues are suffering from abusive supervision; they should also notify themselves of their rights and interests in their work. Additionally, as previous literature on bully has shown that there are many barriers for the victim themselves to confront the perpetrators [57,58], it is highly recommended that coworkers or organizations take action to prevent abusive supervision. For example, coworkers are encouraged to report supervisors’ abusive behaviors to upper-level managers if they have witnessed abusive supervision [22]. Furthermore, it is possible for organizations to set up a mailbox where employees may report supervisors’ abusive supervision anonymously. Last, our findings suggested that fear of external threats strengthens the relationship between supervisors’ trait anger and abusive supervision and, later, the relationship between abusive supervision and subordinates’ CCB. Supervisors’ behaviors are particularly important in a crisis situation. Lebel [1] proposed a positive side of the fear of external threats, as it may enhance open-minded supervisors’ information sharing among organization members and, thus, were constructive to the development of the organization. This provides implications for the importance of supervisors’ behaviors toward subordinates and organizations. In times of uncertainty, supervisors should be trained to keep information transparent to avoid doubts or panic and provide timely support and comfort. Additionally, organizations should lower the detrimental effects of external threats by safeguarding employees’ benefits even in adversity. For example, organizations could introduce new options for teleworking, spread useful stress relief knowledge, and give enough dismissal compensation and treat employees with respect and dignity when having layoffs. These help to calm employee worries, prevent the aggravation of abusive supervision and, in turn, prevent higher levels of employee burnout and CCB occurrence [40,59].

### 5.3. Limitations and Future Directions

The present study has some limitations that can provide directions for future studies. First, we used a time-lagged design, which reduces common method issues [60], although it cannot ensure causality. For example, our study found that abusive supervision mediated the positive relationship between supervisors’ trait anger and subordinates’ burnout and CCB. However, it is also possible that subordinates’ burnout leads to higher levels of abusive supervision because supervisors who are worried about the survival or development of the organizations may expect higher levels of work efforts from subordinates [61]. When they perceive subordinates’ burnout or reluctance to do work, they may engage in more abusive supervision. It is also possible that subordinates’ CCB response to abusive supervision further leads to higher levels of burnout as CCB is resource-consuming [2]. Therefore, future studies can use experimental designs, for example, by manipulating participants’ fear of external threats to investigate how fear plays a role in their actions or reactions. Additionally, future studies could adopt a more rigid longitudinal design and cross-lag analyses to determine the sequence of the variables.

Furthermore, the current study examined burnout as one of the outcomes. However, the results showed that subordinates with higher levels of fear of external threats did not burn out more than those at lower levels. It is possible that subordinates may have more severe negative emotional reactions, such as anxiety and depression, than burnout [21]. For subordinates who are worried about losing their employment or who have witnessed others losing their jobs, they may value their current job more than ever. Future studies can investigate outcomes such as negative emotions to investigate whether their fear of external threats exacerbates the positive relationship of abusive supervision with negative emotions. In addition, future studies could also measure subordinates’ attitudes toward their current work to examine whether there is a positive relationship between their fear of external threats and the value of their work.

Lastly, our study was conducted in China, a typical Asian country with high levels of power distance, a collectivistic culture, and a high-performance orientation [62]. As noted by Salin et al. [62], employees in different countries would perceive and react to abusive supervision differently. It is possible that supervisors in China enact more abusive supervision when faced up with external threats because employees in high power distance and high performance-oriented culture report that supervisors are more likely to misuse their power. Additionally, subordinates in high-power distant countries may react to abusive supervision with more CCB because they are not encouraged to disobey their supervisors. Future studies can explore the roles culture plays in the proposed relationships. 

## 6. Conclusions

Our study highlights that, in an era of uncertainty, both supervisors’ and subordinates’ fear of external threats influences their workplace behaviors. Specifically, we discovered that supervisors’ fear of external threats strengthens the positive relationship between trait anger and abusive supervision, whereas subordinates’ fear of external threats strengthens subordinates’ CCB as a response to abusive supervision. Generally, our study finds that fear of external threats may be an important situational factor that influences both supervisors’ and subordinates’ behaviors and highlights that proactive actions such as positive communication skills and supportive leadership training should be taken to help employees adapt to the uncertainty and reduce excessive fear of external threats. 

## Figures and Tables

**Table 1:** Results of Confirmatory Factor Analysis for the Variable Measures.

Model	χ^2^ (*df*)	RMSEA	CFI	TLI	Change from Hypothesized Model
					∆χ^2^ (*df*)
1. Six-factor model	996.17 ** (604)	0.05	0.95	0.94	
2. Five-factor model (two outcomes were combined into one factor)	2016.77 ** (612)	0.08	0.81	0.80	1020.60 ** (8)
3. Four-factor model (abusive supervision and trait anger were combined into one factor; two outcomes were combined into one factor)	2545.55 ** (619)	0.10	0.74	0.72	1549.38 ** (15)
4. Two-factor model (variables rated by supervisors and subordinates were combined into one factor, respectively)	2784.37 ** (626)	0.11	0.71	0.69	1788.20 ** (22)
5. One-factor model (all items were loaded onto one factor)	3609.12 ** (629)	0.12	0.60	0.58	2612.95 ** (25)

*N* = 322 dyads. *df* = degrees of freedom; CFI = comparative fit index; TLI = Tucker–Lewis index; RMSEA = root mean square error of approximation. Significance was determined using a two-tailed test. ** *p* < 0.01.

**Table 2:** Means, Standard Deviations, and Correlations of Variables.

	*M*	*SD*	1	2	3	4	5	6	7	8	9	10
1. Supervisor age	36.21	7.51										
2. Supervisor gender ^a^	1.41	0.49	0.10									
3. Subordinate age	27.93	7.01	0.02	0.10								
4. Subordinate gender ^b^	1.57	0.50	0.09	−0.02	−0.06							
5. Trait anger	1.89	0.82	−0.09	0.07	−0.02	−0.02	(0.90)					
6. Supervisors’ fear of external threats	3.22	0.69	−0.03	0.12 *	−0.07	0.06	−0.01	(0.85)				
7. Abusive supervision	1.91	0.61	0.01	0.02	0.04	−0.06	0.41 **	0.08	(0.89)			
8. Subordinates’ fear of external threats	3.04	1.04	−0.03	0.06	−0.07	−0.05	0.21 **	0.43 **	0.05	(0.82)		
9. Burnout	3.23	0.68	0.05	0.19 **	0.04	0.10	0.51 **	0.12 **	0.42 **	0.12 *	(0.89)	
10. CCB	2.62	0.87	0.01	0.01	0.10	0.18 **	0.23 **	0.10**	0.31**	0.10	0.06	(0.88)

*N* = 322 dyads. ^a^: 1 = male. ^b^: 1 = male. CCB = compulsory citizenship behaviors. Alpha internal consistency reliability coefficients appear on the main diagonal. Significance was determined using a two-tailed test. ^a^ 1 = male, 2 = female. * *p* < 0.05, ** *p* < 0.01.

**Table 3:** Moderation and Moderated Mediation Effects.

Variable	Model 1		Model 2				Model 3			
	Abusive Supervision		Burnout		CCB		Burnout		CCB	
	*β*	*SE*	*β*	*SE*	*β*	*SE*	*β*	*SE*	*β*	*SE*
Supervisor age	0.01	0.01	0.06	0.04	0.03	0.05	0.01	0.01	0.01	0.01
Supervisor gender ^a^	−0.06	0.06	0.16 **	0.04	−0.01	0.05	0.01	0.01	0.02	0.10
Subordinate age	0.01	0.01	0.03	0.04	0.08	0.05	0.02	0.05	0.01	0.01
Subordinates gender ^b^	−0.10	0.06	0.10 *	0.04	0.17 *	0.05	0.01	0.01	0.31 *	0.09
Dyadic tenure	−0.03	0.02	0.06	0.05	0.01	0.03	0.01	0.02	0.01	0.03
Trait anger	−0.31	0.16	0.39 **	0.06	0.16 *	0.06	0.29 **	0.04	0.12 *	0.06
Supervisors’ fear of external threats	−0.38 **	0.10								
Trait anger * supervisors’ fear of external threats	0.20 **	0.05								
Abusive supervision			0.27 **	0.06	0.34 **	0.09	0.16	0.17	−0.12	0.12
Subordinates’ fear of external threats							0.04	0.14	−0.20	0.15
Abusive supervision * Subordinates’ fear of external threats							0.03	0.07	0.23 **	0.07
*R* ^2^	0.22 **	0.04	0.37 **	0.05	0.14 **	0.05	0.45 **	0.08	0.30 **	0.04

Note: *N* = 322 dyads. ^a^: 1 = male. ^b^: 1 = male. Bootstrapping = 10,000. Significance was determined using a two-tailed test. ^a^ 1 = male, 2 = female. * *p* < 0.05, ** *p* < 0.01.

## Data Availability

The main data can be received upon a reasonable request from the authors.
